# Relationships between the resting-state network and the P3: Evidence from a scalp EEG study

**DOI:** 10.1038/srep15129

**Published:** 2015-10-12

**Authors:** Fali Li, Tiejun Liu, Fei Wang, He Li, Diankun Gong, Rui Zhang, Yi Jiang, Yin Tian, Daqing Guo, Dezhong Yao, Peng Xu

**Affiliations:** 1 Key Laboratory for NeuroInformation of Ministry of Education, School of Life Science and Technology, University of Electronic Science and Technology of China, Chengdu, 610054, China; 2 Center for Information in BioMedicine, University of Electronic Science and Technology of China, Chengdu, 610054, China; 3 College of Bio-information, ChongQing University of Posts and Telecommunications, Chongqing, 400065, China

## Abstract

The P3 is an important event-related potential that can be used to identify neural activity related to the cognitive processes of the human brain. However, the relationships, especially the functional correlations, between resting-state brain activity and the P3 have not been well established. In this study, we investigated the relationships between P3 properties (i.e., amplitude and latency) and resting-state brain networks. The results indicated that P3 amplitude was significantly correlated with resting-state network topology, and in general, larger P3 amplitudes could be evoked when the resting-state brain network was more efficient. However, no significant relationships were found for the corresponding P3 latency. Additionally, the long-range connections between the prefrontal/frontal and parietal/occipital brain regions, which represent the synchronous activity of these areas, were functionally related to the P3 parameters, especially P3 amplitude. The findings of the current study may help us better understand inter-subject variation in the P3, which may be instructive for clinical diagnosis, cognitive neuroscience studies, and potential subject selection for brain-computer interface applications.

The P3 is one of the most important event-related potentials (ERPs)^1^ that can be elicited by the oddball paradigm. In the traditional oddball paradigm, two types of stimuli, target and standard stimuli, are presented to subjects in a random order. During the experimental task, the subject is required to focus his or her attention and to discriminate between the presented stimuli by reacting to the target stimuli (counting or pressing a button) while not responding to the standard stimuli. To evoke a strong P3, the target stimulus probability should typically be lower than 20% because target probability strongly influences P3 amplitude and latency^2^,^3^. P3 amplitude is defined as the largest positive peak of the ERP waveform within the time window of 300–500 ms, and the corresponding latency is defined as the time interval from stimulus onset to the point of maximum positive amplitude within the same time window^4^. P3 potentials can reflect various cognition-related brain functions, such as attention allocations and working memory^5^,^6^,^7^. Thus, the P3 can be regarded as a potential biomarker to evaluate a subject’s processing capacity in an experimental task; accordingly, the P3 is widely used in different applications such as clinical diagnosis^4^,^8^,^9^, cognitive neuroscience, and brain-computer interfaces (BCIs)^10^,^11^.

However, P3 amplitude and latency vary among subjects. An earlier report by Ou *et al.* revealed that a larger P3 amplitude was acquired over different brain regions from poor navigators in driving tasks^12^. A BCI study based on the P3 also indicated that there was cross-subject variability in the P3 amplitude based on performance^13^. Thus, it is important to identify the underlying neural mechanisms that can account for the variation in P3 potentials among humans, and various studies have been conducted with this aim^14^,^15^,^16^. Studies that utilized electroencephalography (EEG), functional magnetic resonance imaging (fMRI) or their combination all showed that the P3 was correlated with the activity of multiple brain cortices^14^,^16^,^17^,^18^. An experiment conducted by Horovitz*. et al.* also reported the considerable contribution of multiple regions (i.e., the thalamus and insula) to P3 amplitude based on the close relationships between P3 amplitude and changes in blood oxygenation level-dependent signals^15^.

The majority of previous studies have focused on focal regions where the P3 is elicited; few studies have considered the interactions among multiple brain regions^14^,^19^. The brain functions as a complex network, with interactions among various brain cortices. Cognition-related brain functions, such as attention and working memory, involve interactions between specialized brain regions that are spatially distributed but functionally linked^20^,^21^. In recent decades, network analysis has become an increasingly important part of the study of human brain function. Brain network efficiency is strongly related to human intelligence^22^. The dynamic interactions of distributed brain areas in large-scale networks, rather than the isolated operations of these regions, greatly contribute to human cognition^23^. Studies of P3 topology have also demonstrated the importance of various neural networks for the P3^24^.

However, these studies have mainly concentrated on task-related networks without considering the relationship between the resting-state network and the P3. During the resting state, the brain is still activated; this pattern of activation is characterized by a specific network mode^25^, and some of the related neuronal activity is associated with specific brain functions^26^,^27^,^28^,^29^, as confirmed by convergent evidence from various resting-state-network-based studies^30^,^31^. Similar to the relationship between background processing networks and steady-state visual evoked potentials (SSVEPs)^32^,^33^, resting-state brain activity is also correlated with the P3^34^,^35^,^36^. Although the findings from three related experiments conducted by Polich, which concentrated on the relationship between EEG parameters (spectral power and mean frequency) and P3 properties, consistently demonstrated that scalp EEG variations significantly contributed to individual variations in the P3^35^, the related resting-state network analysis has rarely been considered^34^,^36^,^37^. Thus, in the current study, we investigated the relationships between the resting-state network and the P3 to deepen our understanding of the P3.

## Results

### P3 amplitude and latency

P3 amplitude and latency varied across subjects, with a mean P3 amplitude of 4.27 ± 1.14 μv and a mean P3 latency of 401.44 ± 34.16 ms.

When the calculations of P3 amplitude and latency were completed, Pearson’s correlation was used to compare P3 amplitude with the corresponding latency. The correlation analysis indicated that there was an insignificant negative relationship between P3 amplitude and the corresponding latency (*r *= −0.22, *p *> 0.05).

### The relationships between P3 amplitude/latency and mean functional connectivity (*MFC*)

**Figure 1:**
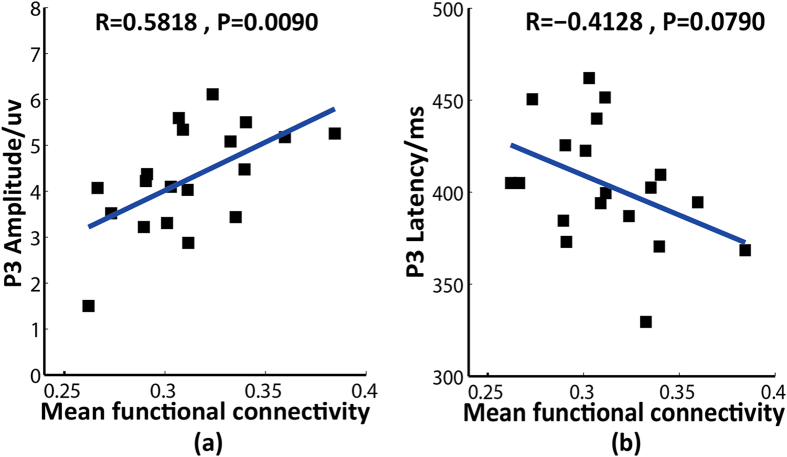
Relationships between the mean functional connectivity and P3 amplitude/latency. (**a**) The mean functional connectivity vs. P3 amplitude. (**b**) The mean functional connectivity vs. P3 latency. In each sub-figure, the blue line is the fitted curve; R indicates the correlation coefficient, and P indicates statistical significance.

The *MFC* reflects the synchronization level of brain activity. The relationships between *MFC* and the P3 properties are shown in Fig. 1. The *MFC* was positively correlated with P3 amplitude (*r *= 0.582, *p *< 0.01) but was insignificantly negatively correlated with P3 latency (*r *= −0.413, *p *> 0.05).

### The relationships between P3 amplitude/latency and resting-state network properties

**Figure 2:**
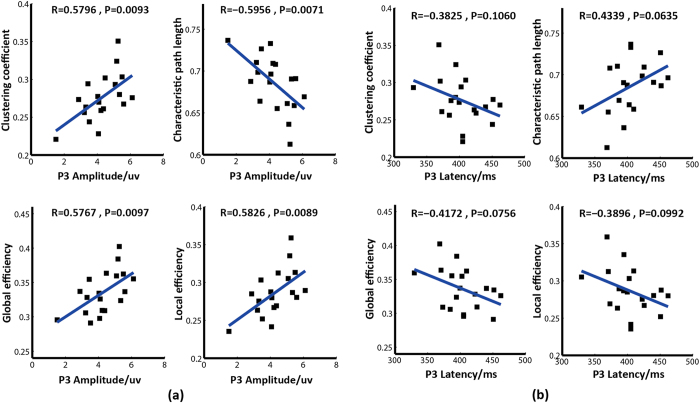
Relationships between the network properties and P3 amplitude/latency. (**a**) Correlations between the resting-state network properties and P3 amplitude. (**b**) Correlations between the resting-state network properties and P3 latency. In each sub-figure, the blue line is the fitted curve; R indicates the correlation coefficient, and P indicates statistical significance.

The clustering coefficient (*C)*, global efficiency (*Ge)*, local efficiency (*Le)*, and characteristic path length (*L)* are four network properties that depict ability of the resting-state network to process and transfer information. The relationships between the four network properties and the P3 properties are shown in Fig. 2. The scatter plots in Fig. 2 indicate that the *C*, *Ge*, and *Le* were significantly positively correlated with P3 amplitude (*p *< 0.01). In contrast, the *L* was negatively correlated with P3 amplitude (*p *< 0.01). However, the network properties exhibited insignificant opposite relationships with the corresponding P3 latency (*p *> 0.05) that were in the opposite direction of the relationships between the network properties and P3 amplitude.

**Figure 3:**
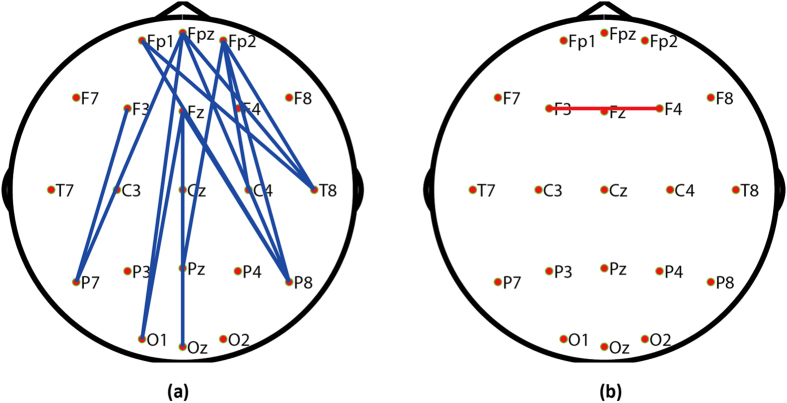
Topography of the edges whose strengths were significantly related to P3 amplitude and the corresponding latency. (**a**) Edges that were significantly correlated with P3 amplitude (p < 0.01). (**b**) Edges that were significantly correlated with P3 latency (p < 0.01). In each sub-figure, blue edges indicate positive correlations between two variables, and red edges represent negative correlations between two variables.

Edge strength represents the exchange of information between two nodes. To further determine which node linkages affected the P3, the correlations between the P3 properties and each network edge were calculated. The spatial distribution of the edges that were significantly correlated (*p *< 0.01) with P3 amplitude and latency is provided in Fig. 3.

## Discussion

Various studies have demonstrated that the P3 involves multiple brain areas, including loci in the temporal, frontal, and parietal lobes^4^,^16^,^24^,^38^. Constructed networks containing these regions have been found to be correlated with higher-order cognitive processes, such as attention, intelligence, and working memory, and simultaneous interactions between these regions contribute to the P3^4^,^39^. People with cognitive deficits due to mental and neurological diseases showed longer P3 latencies and smaller P3 amplitudes^40^. Lesions to the grey and white matter of the frontal and temporo-parietal junction due to hazardous substances resulted in P3 amplitude abnormalities^17^,^18^. These existing studies converge to suggest that the P3 represents a complex summation of activity from the interaction of multiple brain regions, particularly the association areas of the cerebral cortex^24^,^41^. This is consistent with the recent finding that multiple levels of cognitive processes were modulated by the patterns of brain networks that dynamically shifted between integration and separation across multiple network nodes^42^,^43^,^44^. Moreover, other studies have also demonstrated strong correlations between the resting-state network and several physiological behaviors, including strategy choices^27^, reaction time in Go/No-go tasks^29^, and SSVEPs^32^. In the current study, we assumed that the P3 parameters and the network properties of the constructed networks could reflect the neural mechanisms of cognitive processing from different aspects, and these networks and the P3 might be strongly related. Therefore, we examined the potential relationships between the P3 and resting-state EEG networks and aimed to identify the underlying neural mechanism of the P3 based on the resting-state network.

P3 amplitude and latency varied widely across subjects. Additionally, P3 amplitude was insignificantly negatively correlated with the corresponding latency across all subjects (*p *> 0.05). In our study, P3 amplitude and latency were extracted from five electrodes (CPz, CP1, CP2, Cz, and Pz) that were close to the parietal region. P3 amplitude and latency represent the strength and timing of cognitive processing, respectively^6^,^45^. Specifically, P3 amplitude is correlated with the amount of attentional resources devoted to a specific task, while P3 latency is regarded as an indicator of brain information processing efficiency^46^. A shorter P3 latency indicates increased cognitive processing efficiency, which would result in increased P3 amplitude^47^. Compared with P3 amplitude, which was estimated using an average value to reduce the influence of noise, P3 latency is more likely to be influenced by noise, which results in an inaccurate estimation. Therefore, the negative relationship between P3 amplitude and the corresponding latency was not significant. And the inaccurate estimation of P3 latency could also account for the finding that P3 latency was insignificantly negatively correlated with the resting-state network properties.

Figure 1 demonstrates that the synchrony of the resting-state brain is functionally related to a strong and efficient P3, especially P3 amplitude. The increased *MFC* indicated that the related brain areas were closely linked, which may have resulted in increased efficiency of information processing and transfer for higher-order cognitive functions.

The scatter plots in Fig. 2 indicate that the increased P3 amplitude was accompanied by an increased *C*, *Ge*, and *Le* as well as a shorter *L*. The corresponding relationships with P3 latency were the opposite of those with P3 amplitude and were not significant (*p *> 0.05). Because an increased *C*, *Ge*, and *Le* as well as a shorter *L* represented an increase in the efficiency of information processing in our brain network, the relationships in Fig. 2 indicate that an efficient resting-state network corresponded to a strong P3. The P3 has been regarded as a biomarker for higher-order cognitive processes such as attention, working memory, and intelligence, and these functions are also closely related to network topologies^22^,^23^. Because no external stimulus is imposed, the resting-state brain network can reflect the fundamental linkage structure of related areas, which determines whether the network can efficiently process information. In addition, higher-order cognition involves complex information processing. Thus, when a resting-state network is highly efficient, it can potentially provide more efficient information processing during a task, which results in a P3 with a strong amplitude and short latency.

As shown in Fig. 3, the edges whose strengths were significantly correlated with P3 amplitude were mainly the long-range connections between the prefrontal/frontal cortex and parietal/occipital region (Fig. 3(a)); in contrast, very few edges were strongly correlated with P3 latency (Fig. 3(b)). The P3 results from cognition-related brain information processing, such as attention, intelligence, and working memory, which require the interaction among large-scale brain areas^23^,^48^. Studies have demonstrated that synchronization between anterior and posterior regions plays an important role in information processing and transfer in cognitive tasks^49^, e.g., studies of both rats and humans have consistently demonstrated that long-range connections between frontal and occipital regions facilitate the elicitation of SSVEPs^32^,^33^. Even in the non-task-related resting state, prefrontal, frontal, and occipital areas were still recruited by some specific cognitive processes^50^,^51^. The frontal and parietal cortices were shown to be crucial for top-down/bottom-up attentional control^52^, and working memory also required the interaction of the prefrontal and parietal cortices^53^. Additionally, in a study of the origins of the P3 based on source separation using source localization methods, Zhang *et al.* reported the contribution of synchronized brain activity between the anterior and posterior brain regions to the generation of the P3^24^. Additionally, the importance of these long-range connections between the prefrontal/frontal cortex and parietal/occipital region for the P3 was also demonstrated by the significant reduction in P3 amplitude of frontal-lobe lesioned patients^20^.

Another finding of our study was that although consistent results were identified for the relationships between the network indexes (network properties, edge strength, and *MFC*) and the two P3 properties (amplitude and latency), differences remained for the two P3 properties. For instance, P3 amplitude was stably and strongly correlated with the resting-state network, while P3 latency was not. The insignificant correlation found for P3 latency was mainly attributed to inaccurate estimation because the averaging strategy was difficult to apply to extract the latency in the experimental situation.

The aim of this study was to investigate the relationships between resting-state network properties and the P3 to identify the neural mechanisms of the P3 from the perspective of resting-state network. The efficiency of the resting-state brain network was functionally related to the P3 parameters, especially P3 amplitude. Additionally, the prefrontal/frontal and parietal/occipital brain regions and their long-range connections played crucial roles in the generation of the P3. However, because the current study utilized an undirected network analysis, it was not possible to definitively demonstrate the direction of information flow among the relevant brain areas or the causality between the P3 and the resting-state network. In the future, we will utilize causal analyses, such as Granger causality, partial directed coherence, or dynamic causality models, to construct directed networks. Another limitation of the current study is that the subjects were all male, and gender differences may have influenced the findings to some degree; therefore, gender differences should be considered in future work.

## Methods

This experiment was approved by the Institution Research Ethics Board of the Key Laboratory of NeuroInformation of Ministry of Education at University of Electronic Science and Technology of China (UESTC). The methods were also performed according to the guidelines approved by the Institutional Review Board of the Key Laboratory of NeuroInformation of Ministry of Education at UESTC. All participants who took part in our experiment provided written informed consent before the experiment.

### Participants

Twenty-three healthy postgraduates participated in this experiment after providing written informed content. Four participants were discarded from the study because of the failure to acquire reliable resting-state or task-related data without many artifacts. The remaining 19 subjects were right-handed males. The age range was 22–27 years. None of the subjects had used medication, and none of the subjects had a personal or family history of psychiatric or neurological disease.

### Experimental procedures

The subjects were instructed to remain relaxed, to refrain from extensive head motion, and to not move their body during the task. Data recording was initiated with a four-minute resting-state EEG while the subjects’ eyes were closed. After a one-minute break, the subjects performed the P3 task. When the experimental task was completed, the subjects were required to verbally state the number of target stimuli they counted.

**Figure 4:**
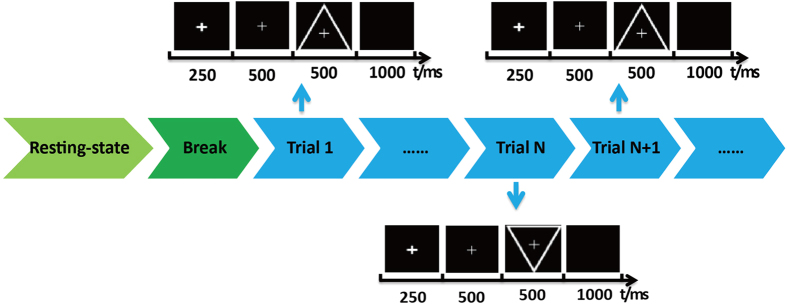
Experimental protocol of the resting-state and experimental tasks.

The detailed procedures of the experimental task are illustrated in Fig. 4; four minutes of resting-state data were recorded prior to the P3 task, followed by a 1-minute break. Then, the task to discriminate between the target/standard stimuli was conducted. At the beginning of each trial, all subjects were asked to fixate on the center of the computer monitor. A bold cross then appeared, which served as a cue for the subjects to concentrate their attention on the monitor without moving their eyes. After 250 ms, a thin cross appeared, informing the subjects a stimulus would subsequently appear. The thin cross was presented for 500 ms, and then a target or standard stimulus was presented for 500 ms. The subjects were asked to pay attention to and count the number of target stimuli and to not react to the standard stimuli. The target stimuli consisted of downward-oriented triangles with thin crosses in their centers, and the standard stimuli consisted of upward-oriented triangles with thin crosses in their centers. After a 1,000-ms break, the next trial was initiated. The target stimuli were randomly presented 20% of the time. The experimental task consisted of 150 trials, with approximately 120 standard stimuli presented and 30 target stimuli presented.

### EEG data recording

The EEG data were recorded with 64 Ag/AgCl electrodes, which were positioned according to the extended 10/20 system and digitized with a sampling rate of 500 Hz (Brain Products GmbH). The FCz and AFz electrodes served as the reference and ground, respectively. Vertical and horizontal electrooculogram (EOG) data were recorded from two additional channels to monitor eye movements. The impedance for all electrodes was maintained below 5 KΩ, and the online filter band was 0.01–100 Hz.

### EEG data analysis

**Figure 5:**
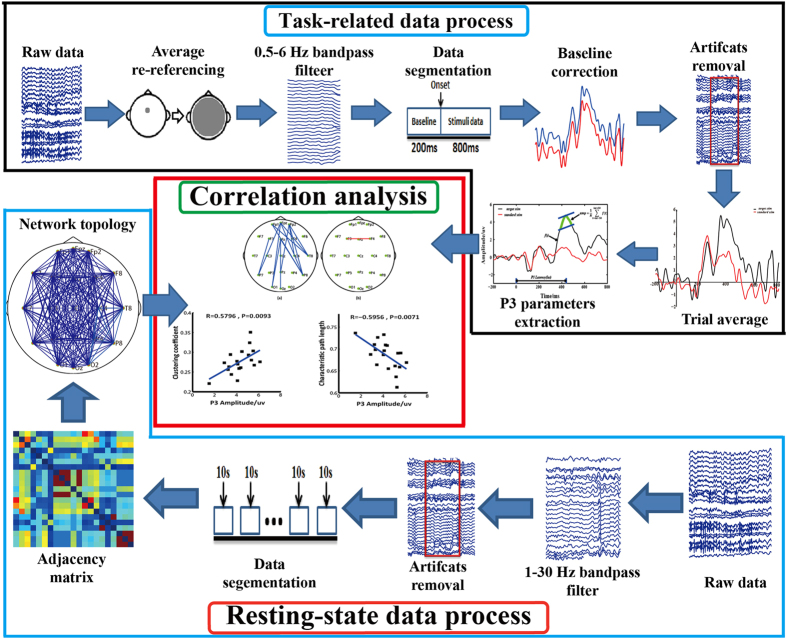
Analysis procedure of the task-related and resting-state data. P3 amplitude and latency were extracted from the task-related data, whereas the network topologies were calculated from the resting-state data. A correlation analysis was subsequently performed.

The EEG recordings consisted of resting-state and task-related EEGs. The aim of the task-related EEGs was to reliably estimate the amplitude and latency of the P3, whereas the resting state was used to build the corresponding brain network. Therefore, the EEG data analyses consisted of the analysis of both the P3 waveform and the resting-state EEG data. The corresponding analysis was carried out using Matlab 2014a (The MathWorks Inc.), and the analysis procedure is depicted in Fig. 5. Detailed information regarding the data processing is presented in the following sections.

### Extraction of P3 amplitude and latency

**Figure 6:**
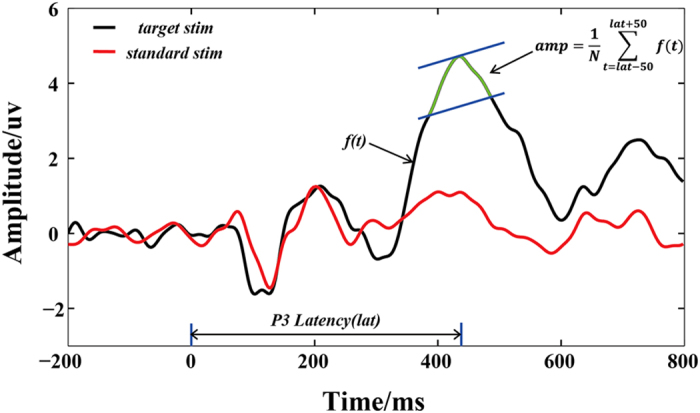
Definitions of P3 amplitude and the corresponding latency. The P3 amplitude represents the mean value of 51 sample points, whereas the P3 latency represents the time interval between stimulus onset and the largest positive P3 peak.

For the task-related EEGs, the pre-processing included average re-referencing, 0.5- to 6-Hz bandpass filtering^54^, data segmentation, baseline correction, and the exclusion of artifact-containing trials (±75 μv was the threshold for ocular artifacts). After pre-processing, an average for the two types of ERPs (i.e., standard and target) was obtained according to the labels for each subject. P3 amplitude and latency were subsequently estimated using the averaged target ERP. To obtain a reliable estimate of P3 amplitude and latency, five electrodes (i.e., CPz, CP1, CP2, Cz, and Pz) that were located in posterior areas and that consistently exhibited visually obvious P3s were selected. Considering the effect of noise, P3 amplitude was defined as the average amplitude in the time window ±50 ms, with the largest positive P3 peak at the center; the corresponding latency was the time interval between stimulus onset and the largest positive P3 peak. The method used to determine of P3 amplitude and latency is illustrated in Fig. 6.

For each subject, the corresponding P3 amplitudes and latencies were estimated for electrodes CPz, CP1, CP2, Cz, and Pz, and the averaged values across the five electrodes were treated as the P3 amplitude and latency for each individual subject.

### Resting-state network analysis

#### Adjacency (connectivity) matrix estimation

To reduce the effect of volume conduction, the 21 canonical electrodes of the 10–20 system were selected from the 64 electrodes to construct the brain network^55^.

The eyes-closed, resting-state EEG signals were bandpass filtered within 1–30 Hz and continuously divided into 10-second long segments. The segments were then visually checked to remove those with ocular or head movement artifacts. Approximately 10 segments were further analyzed for each subject.

Based on the 10-second long segments, the coherence (*Coh*) was used as a measure of the interactions between two electrodes. *Coh* is the most commonly used method of analyzing cooperative, synchrony-defined cortical neuronal assemblies, and represents the linear relationship at a specific frequency between two signals, *x(t*) and *y(t)*, based on their cross-spectrum^56^. *Coh* is expressed as follows:


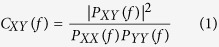


where *P*_*XY*_*(f )* is the cross-spectrum of *x(t)* and *y(t)* at frequency *f*, and *P*_*xx*_*(f )* and *P*_*yy*_*(f )* indicate the auto spectrum at frequency *f*, as estimated from the Welch-based spectrum. Based on the frequency-dependent *Coh*, *C*_*XY*_*(f )*, the edge linkages were estimated by averaging the *Coh* strength within the whole frequency band. Following the calculation of the paired *Coh* between each pair of 21 electrodes, the 21 × 21 weighted adjacency (connectivity) matrix was constructed to represent the interactions among the 21 nodes for each segment of each individual subject. The adjacency (connectivity) matrices were then averaged across segments to achieve the final adjacency matrix for each subject. Finally, the brain network was constructed based on the 21 × 21 weighted adjacency (connectivity) matrix using the corresponding *Coh* as the edge linkage, 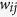, between two nodes, *i* and *j*.

### Network properties

After the weighted network was constructed, the related resting-state network indexes were calculated using graph theories.

Let 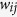 represent the connection strength between node *i* and node *j*, 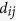 represent the *L* between node *i* and node *j*, *N* represent the node number, and 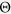 represent the set of all nodes of a resting-state network.

*C* is defined as the fraction of triangles around an individual network node, and *Le* is defined as the average efficiency of the local subgraphs. They both relate to the estimation of the potential for functional segregation between brain areas. Therefore, they consistently reflect the local information processing capacity of human brain networks and can be defined as follows:


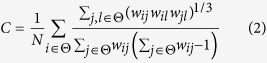



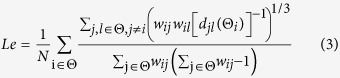


*Ge* is defined as the average efficiency of the related brain network, and *L* is defined as the mean value of the shortest path length between all pairs of network nodes. Both are applied to estimate the potential for functional integration between brain regions. Thus, they consistently represent the efficiency of global information processing of human brain networks, and are calculated as follows:


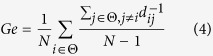



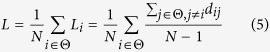


The *MFC* is related to the mean value of all of the existing connections between each pair of nodes, which was simultaneously defined as


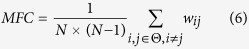


Previous studies provide details regarding the graph analysis^57^.

### Correlation analysis between P3 properties and network properties

Pearson’s correlation analysis was applied to the P3 properties and the resting-state EEG network properties to identify potential relationships across subjects^33^.

## Additional Information

**How to cite this article**: Li, F. *et al.* Relationships between the resting-state network and the P3: Evidence from a scalp EEG study. *Sci. Rep.* **5**, 15129; doi: 10.1038/srep15129 (2015).
